# Surface Effects and Optical Properties of Self-Assembled Nanostructured a-Si:Al

**DOI:** 10.3390/nano9081106

**Published:** 2019-08-01

**Authors:** Torunn Kjeldstad, Annett Thøgersen, Marit Stange, Ingvild Thue Jensen, Eduard Monakhov, Augustinas Galeckas

**Affiliations:** 1 Department of Physics/Centre for Materials Science and Nanotechnology, University of Oslo, P.O. Box 1048 Blindern, N-0316 Oslo, Norway; 2 SINTEF Industry, P.O. Box 124 Blindern, 0314 Oslo, Norway

**Keywords:** amorphous silicon, self-assembly, aluminium, nanowires, nanoporous, photoluminescence, vibronic modes

## Abstract

We present a study of the surface effects and optical properties of the self-assembled nanostructures comprised of vertically aligned 5 nm-diameter Al nanowires embedded in an amorphous Si matrix (a-Si:Al). The controlled (partial) removal of Al nanowires in a selective etching process yielded nanoporous a-Si media with a variable effective surface area. Different spectroscopy techniques, such as X-ray photoelectron spectroscopy (XPS), UV-Vis spectrophotometry and photoluminescence (PL), have been combined to investigate the impact of such nanostructuring on optical absorption and emission properties. We also examine long-term exposure to air ambient and show that increasing level of surface oxidation determines the oxide defect-related nature of the dominant PL emission from the nanoporous structures. The role of bulk, nanosize and surface effects in optical properties has been separated and quantified, providing a better understanding of the potential of such nanoporous a-Si:Al structures for future device developments.

## 1. Introduction

Silicon (Si) nanostructures are the focus of extensive research activities seeking to circumvent the fundamental limitation set by the indirect bandgap of Si that hinders many potential applications, particularly in optoelectronics. A variety of Si nanostructures, such as porous Si, nanowalls, nanoholes, and nanowires (NWs) have achieved quantum confinement and increased optical absorption in Si [1,2,3,4]. The significant developments in Si nanostructuring demonstrated in recent years also indicate a gradual shift of focus from nanosize-related (quantum confinement) phenomena to surface/interface-related effects, including a variety of interactions with adsorbents, impurities, defects, etc. [5,6,7,8]. The important discoveries in this regard are in the field of surface functionalization for switching the bandgap type to direct [9,10,11].

The quantum confinement and surface-related effects apply equally to the amorphous silicon (a-Si) material system. The nanostructuring of a-Si can be realized, for instance, by introducing a high-density nanopore network, as will be demonstrated in the present work. Such a nanoporous a-Si potentially represents a complementary constituent for all-Si based optical communication and can also be envisaged as an emerging material in gas and biomedical sensing. Indeed, the upsurge of surface-to-bulk ratio in nanoporous media makes surface-related effects the dominating factor in light interaction with matter, directly affecting optical scattering (Rayleigh and Mie), absorption and emission properties, and thus providing unique opportunities for novel optoelectronic and sensor applications.

In contrast to porous crystalline Si (c-Si), which is a well-established system with extensively studied pore-size dependent luminescence properties [12,13], there are only scarce reports on porous a-Si, not least because the fabrication processes have proved to be much more challenging [14,15]. The porous a-Si material can be formed through anodization of a-Si [15,16], which generally yields a branched arrangement of random size pores. On the other hand, much finer and denser porosity on a nanoscale can be realized in co-sputtered a-Si:Al material system via a two-step process that involves Al and Si phase separation and subsequent selective etching of Al [17,18,19]. This method exploits the fact that at certain growth conditions the low solid-solubility of Al in Si leads to the self-assembled formation of Al NWs, yielding a densely packed network of vertically aligned NWs (with typical diameter and separation pitch of ~5 nm) embedded in the a-Si matrix. The Al NWs are then removed by selective chemical etching, leaving behind an orderly arrangement of hollow channels with uniform size distribution. This kind of well-ordered nanoporous a-Si provides a universal platform for investigating both nanosize-related and surface-related effects, as well as a template suitable for more complex future developments, such as coaxial core-shell or p-n junction structures. However, many physical aspects that determine and/or limit the practicability of such novel applications are still not fully understood. The large effective surface area of a nanoporous structure comprised of vertically aligned tubular voids in a-Si implies an intricate interaction of surface-related effects and those representing bulk material properties, possibly influenced by quantum confinement effects in localized (inter-tubular) regions. The processes of natural surface oxidation and adsorption inside the nanopores at ambient air conditions are therefore of paramount importance, especially in view of the eminent optical activity of oxidized Si surfaces associated with adsorbed species on the oxide surface, defects within the oxide and at the Si and oxide interface [13,20,21].

We have previously shown that removal of the Al nanowires may be monitored by relatively simple optical reflectance measurements, which allows for controlling the fabrication process on a macroscopic scale [22]. As a continued step towards utilizing the nanoporous a-Si:Al in future applications, we here investigate optical emission and absorption properties, as well as long-term stability. We attempt to unravel the optical properties of these nanostructures by exploring and correlating the underlying phenomena on the nanoscale by combining the surface sensitive techniques X-ray photoelectron spectroscopy (XPS) and photoluminescence (PL) measurements, in addition to measuring and modeling reflectance data.

## 2. Materials and Methods

The nanostructured samples were produced by co-sputtering of Si and Al by a CVC 601 magnetron sputter system onto single crystalline p-Si (100) substrates. The deposition was performed at room temperature by alternating thin layers with 40 at.% Al and 60 at.% Si ratios obtained by using powers of 400 W for Si and 150 W for Al, 2.5 rpm and 22 min sputtering time, resulting in a 100 nm thick film. The sputtering process was carried out at 3 mTorr sputtering pressure using a continuous flow of argon and hydrogen (Ar + 13% H_2_). The self-assembled Al nanowires were removed from the a-Si matrix by wet etching in a 1:1 solution of 37% HCl in deionized water. After the etching procedure, the nanoporous samples were rinsed in deionized water. More details on the nanostructuring of a-Si:Al films can be found in the work by Thøgersen et al. [19].

**Figure 1:**
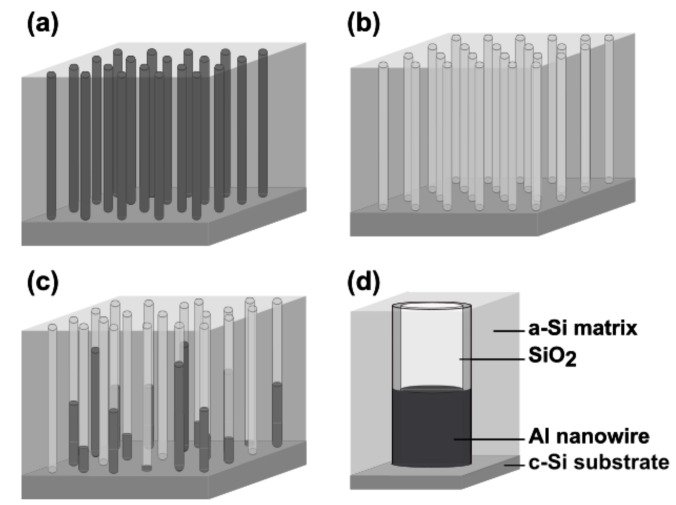
Schematic illustration of the investigated nanostructures: (**a**) As-deposited a-Si:Al with self-assembled Al nanowires (NWs), (**b**) nanoporous a-Si after complete removal of Al NWs, (**c**) nanoporous a-Si:Al with partially-etched Al NWs, (**d**) composition and internal structure of a single partially-etched nanopore.

The samples investigated in this work exemplify three representative stages of a-Si:Al nanostructuring—non-etched (as-deposited film with Al NWs intact), partially-etched, and fully-etched (nanoporous) structures, as illustrated schematically in Figure 1. These samples can be considered as a representation of the possible film compositions for future applications. The long-term stability and interrelated surface effects (natural oxidation and adsorption/desorption) were assessed by examining nanostructures after exposure to air ambient for one week (fully-etched nanoporous structure) and for 34 weeks (partially-etched nanostructure), respectively.

XPS was performed in a KRATOS AXIS ULTRADLD instrument (Kratos Analytical Ltd, Manchester, UK) using monochromatic Al Kα radiation (*hv* = 1486.6 eV). The X-ray source was operated at 10 mA and 15 kV, and high-resolution spectra were acquired with a step size of 0.1 eV and pass energy of 40 eV. The spectra were fitted with the computer program CasaXPS (Casa Software Ltd, Teignmouth, UK) [23]. Sputtering of the sample surface was done using an Ar^+^ ion beam of 0.5 kV delivering 100 μA of current on a 3 × 3 mm^2^ area.

The PL measurements were carried out in the temperature range from 10 K to 300 K using two different excitation wavelengths: A 325 nm line from cw-HeCd laser and a 488 nm line from Ar-laser in combination with low-pass filtering (cut-off at 550 nm) of the detected PL signal. The emission was analyzed by an imaging spectrometer system (Horiba iHR320 coupled to Andor LM658M EMCCD, Oxford Instruments, Abingdon, UK) with a spectral resolution below 2 nm. The total reflectance measurements were performed at room temperature in the wavelength range 186–2500 nm using SolidSpec-3700/3700DUV spectrophotometer (Shimadzu, Kyoto, Japan) equipped with an integrating sphere.

## 3. Results and Discussion

### 3.1. Sample Composition and Surface States

**Figure 2:**
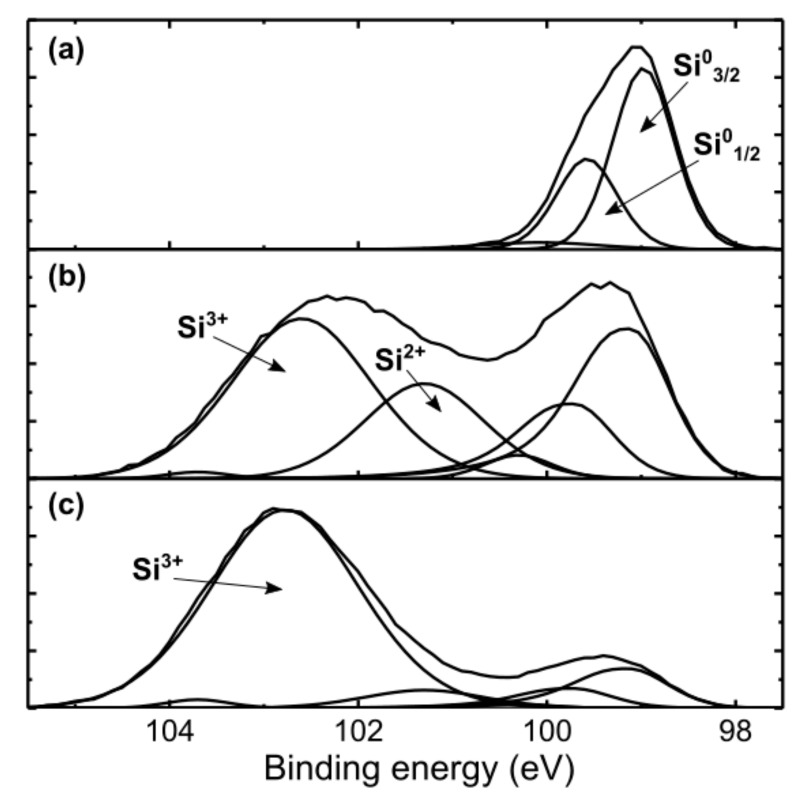
High-resolution XPS spectrum of Si-2p peak measured for (**a**) as-deposited a-Si:Al, (**b**) nanoporous a-Si (one-week exposure to air) and (**c**) nanoporous a-Si:Al (34-week exposure to air).

The initial oxygen content embedded in the as-deposited nanostructured a-Si:Al is low, as shown in our earlier study [19]. The removal of Al NWs leaves behind hollow channels (nanopores) that are prone to surface oxidation once exposed to ambient air, as is also observed for porous c-Si [24]. To evaluate the degree of oxidation over time, etched samples were investigated by XPS after 1 and 34 weeks exposure to air. In order to remove adventitious carbon, 5 min of Ar sputtering was performed before collecting high resolution-spectra. For the etched samples, Ar sputtering is complicated by the hollow channel structure of the samples. However, this should not affect the comparability between similarly structured samples. Figure 2a shows the binding energies of the Si-2p peak for the nanoporous a-Si samples, compared to as-deposited a-Si:Al. The ratio of the different charge states of Si was estimated by fitting high-resolution XPS spectra with Gaussian functions centered at known bonding energies for the different charge states [24,25,26,27]. For elemental Si, the doublet Si-2p^1/2^ and Si-2p^3/2^ were used, while a single Gaussian was used for the oxide. Si^3+^ dominates the fully oxidized sample, with only 12 at.% of the total Si remaining as elemental Si, while the partly oxidized sample consists of 35 at.% elemental Si and both Si^2+^ and Si^3+^. The Ar sputtering may explain the low values of Si^4+^ present on the surface. It is expected that XPS provides information about the first 10 nm of the porous film, thus the results are not affected by a difference in Al concentration between the two porous samples, as the Al concentration is larger towards the substrate. Generally, XPS results confirm the low oxygen level in the as-deposited a-Si:Al. Removal of Al and subsequent surface exposure to air ambient for one-week results in a decrease of signal from elemental silicon due to surface oxidation. Prolonged surface exposure increases the oxidation and further reduces the remaining elemental Si in the a-Si matrix.

### 3.2. Reflectance as a Function of Al Concentration

**Figure 3:**
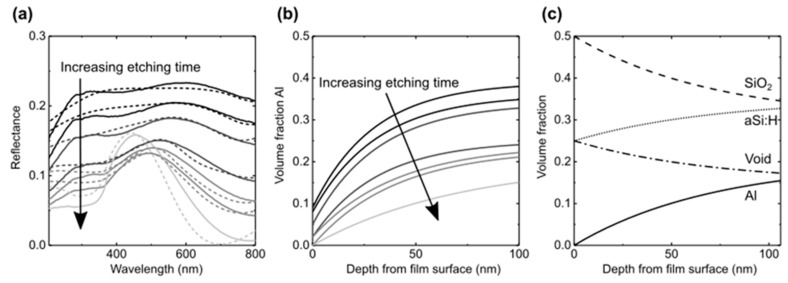
(**a**) Evolution of the total reflectance of a-Si:Al during Al removal (2–18 h of etching) represented by measured (solid lines) and simulated (dotted lines) spectra. (**b**) Depth profiles of Al content within a-Si:Al used for the reflectance calculations. (**c**) Volume fractions of the main constituents as a function of depth used in the reflectance calculations of a-Si:Al etched for 18 h.

Figure 3a shows the evolution of the total reflectance of a-Si:Al film as Al NWs have been gradually etched out. The removal of Al causes a general reduction in reflectance over the visible range as a result of varying in-depth distribution of Al content before interference effects start to dominate while approaching fully nanoporous a-Si. The reflectance during Al removal was modeled by using the transfer-matrix model and approximating the nanostructure to consist of several planar layers with different Al concentrations [28]. The effective refractive index for each layer has been estimated by using the Bruggeman effective medium approximation with a polarization factor of ½ for circular rods [29,30] and known refractive indexes for all components [31,32,33,34]. To estimate the volume fraction, we have assumed that as the Al is removed, half of the remaining elemental silicon is in the form of SiO_2_ and that the expansion of the Si as it oxidizes reduces the void by 50%. This gives volume fractions of *V_SiO_*_2_ = 0.5 − *V_Al_*, *V_aSi_* = 0.25 + *V_Al_*/2, *V_voidi_* = 0.25 − *V_Al_*/2. The dotted line in Figure 3a shows the calculated reflectance based on the Al gradient shown in Figure 3b.

Figure 3c shows the volume fraction of each constituent as a function of depth from the film surface towards the Si substrate. The Al gradient is due to partial etching of the wires, and by controlling this gradient through optimization of etching conditions, the optical properties of the film may be tuned. In addition to reflectance change due to a graded refractive index, plasmonic effects from the Al nanowires may also affect the optical properties of the film. Localized surface plasmon resonances for Al are normally observed in the UV-region, but as the wires are encapsulated in a-Si, these resonances may be shifted into the visible spectrum [35,36]. Thus, fully or partially etched a-Si:Al thin films may be utilized for increased absorption in for example solar cells [37].

### 3.3. Photoluminescence

**Figure 4:**
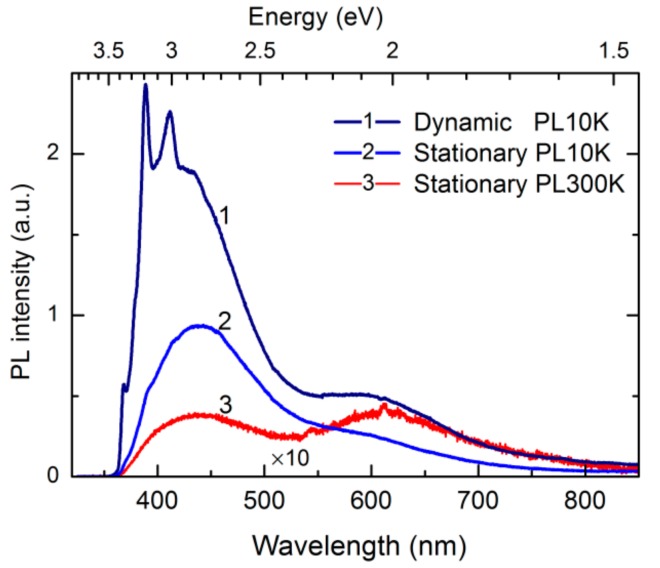
Photoluminescence (PL) spectra of nanostructured a-Si:Al illustrating representative features of emission addressed in this study: Instability under UV irradiation, temperature dependence, and surface effects. Here, spectra labeled Dynamic and Stationary represent two PL acquisition modes: With continuously translated specimen (movable probe position) and conventional probing at a fixed position. Note that the room temperature PL spectrum is magnified by ×10.

Photoluminescence of the nanostructured a-Si:Al and porous a-Si generally comprise several partially overlapping emission bands that stretch throughout the UV and visible range, as illustrated in Figure 4. In the high-energy region of the spectra, a series of sharp features can be observed between 3.0 and 3.4 eV, which appear instable and quench under sustained UV irradiation. The enduring featureless background emission is a broadband centered at around 2.8 eV that dominates PL spectra along with another broad emission component in the visible range (~2 eV). To help distinguish the contributions from different luminescence sources on the surface and in the bulk, the PL measurements were carried out using variable excitation (photon energies 3.8 and 2.5 eV), temperature (10–300 K) and ambient (air and vacuum).

**Figure 5:**
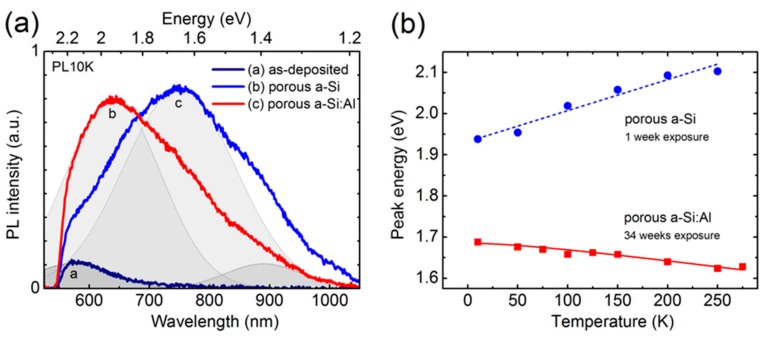
(**a**) PL spectra obtained at 10 K (488 nm excitation and low-pass LP550 filtering) of as-deposited a-Si:Al nanostructure, nanoporous a-Si (fully-etched Al NWs, one-week exposure to air), and porous a-Si:Al (partially-etched Al NWs, 34-week exposure to air). Gaussian curves indicate the main luminescence components. (**b**) PL peak energy as a function of temperature for nanoporous a-Si and porous a-Si:Al. The corresponding temperature coefficients (dE/dT) are +0.75 meV/K and −0.32 meV/K, respectively.

Figure 5a shows PL spectra of the three representative nanostructures obtained at 10 K using 488 nm excitation (2.5 eV) and low-pass filtering with a cut-off at 550 nm wavelength. The nanoporous a-Si and a-Si:Al structures exhibit broad PL bands of similar intensity at 1.65 and 2 eV, respectively, whereas the as-deposited a-Si:Al demonstrates the order of magnitude lower emission at around 2.2 eV. The PL of nanoporous a-Si measured after relatively short-term (one week) natural oxidation appears to represent the bulk properties, i.e., a-Si matrix, as the peak emission at 1.65 eV matches well with the PL signature reported for porous a-Si [38,39]. Further long-term (34 weeks) exposure to natural air and moisture ambient conditions leads to a blue-shift of the emission maximum from 1.65 to 1.95 eV, as can be observed from the PL spectrum of the nanoporous a-Si:Al structure with partially-etched Al NWs. The underlying mechanism behind this apparent shift is the changing mutual contribution from several different luminescence components with fixed spectral positions.

**Figure 6:**
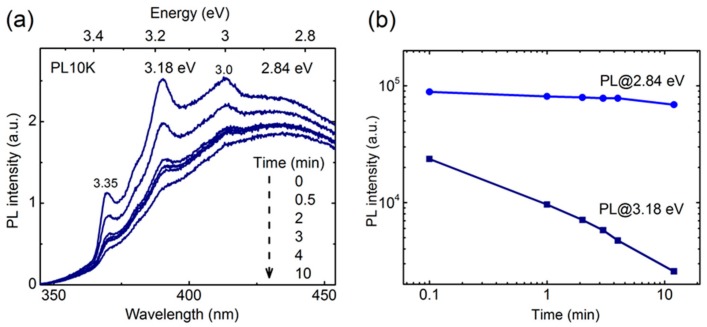
Photoluminescence of nanoporous a-Si:Al (34-week exposure to air): (**a**) Photodegradation of spectral features under UV irradiation revealed by time-lapse PL measurements, (**b**) regression of PL intensity under UV-exposure for the broadband (2.8 eV) and sharp peak (~3.18 eV), respectively.

In Figure 5a, the major emission constituents common to all spectra are indicated by Gaussian curves. It should be noted at this point that our measurements (not shown) of partially-etched a-Si:Al nanostructures with different Al NW content, but similar ambient exposure conditions, demonstrate no change in the peak position, thus ruling out any potential connections of the 0.3 eV blue-shift with residual Al NWs. On the other hand, as shown in Figure 2, the prolonged exposure to air ambient increases surface oxidation and decreases the volume of the remaining a-Si, thus in effect reducing the original spacing between the nanopores in the a-Si framework. According to the models of Wehrspohn and Estes [38,40,41], such an increasing role of spatial confinement within the a-Si matrix may be responsible for the shift of PL emission towards higher energies observed in the present study. This is particularly true for the emission components centered at 1.4 and 1.65 eV (see Figure 5a), which represent respectively bulk and nanoporous, i.e., quantum confinement affected features of the same a-Si matrix [11]. However, this model seems not applicable for the observed 0.3 eV blue-shift of the PL from 1.65 to 1.95 eV, considering the temperature dependencies of the peak positions presented in Figure 5b. Indeed, a distinctive blue-shift of the 1.95 eV peak position with increasing temperature (dE/dT = 0.75 meV/K) suggests that the origin of emission is not pertinent to the a-Si matrix but is rather surface oxide-defect related. By contrast, the 1.65 eV peak demonstrates a red-shift with increasing temperature, which is indicative of temperature-dependent bandgap narrowing (BGN) effect in semiconductors. The experimental data in Figure 6 is fitted with a semi-empirical Varshni expression [42], describing the bandgap variation with temperature as *E_g_*(*T*) = *E*_0_ − *aT*^2^/(*T* + *b*). The estimated BGN parameter *a* = 0.32 meV/K is somewhat smaller than that reported for Si (*a* = 0.47 meV/K), which might be due to counteracting (blue-/red-shift) effect of partially overlapping emission constituents. It is also noteworthy that similar observations reported for a-Si:H [43,44] system further support the present association of 1.65 eV emission with the nanoporous a-Si matrix.

The characteristic emission at around 2 eV is commonly observed from SiO_x_, and several models have been developed to explain the origin of this luminescence. The non-bridging oxygen hole center (NBHOC) is usually considered as the source of 2 eV photoluminescence, but other defects related to oxygen vacancies and E’ type defects have also been suggested [45,46,47,48]. Wolkin et al. proposed that PL pinning observed at 2 eV when Si nanocrystallites were oxidized was a result of emission from trapped excitons on the Si=O bond formed at the interface [49]. The non-stoichiometry of the oxide layer suggests that a variety of defects may be present. Formation of NBHOC is usually associated with radiolysis of hydroxide groups and cleavage of strained Si-O bonds by irradiation. This is supported by previous work, which shows that the intensity of the 2 eV increases with increasing H-content in the sample [46]. Considering that a-Si:Al nanostructures studied in the present work are formed under hydrogen-rich conditions, the presence of a large fraction of hydroxide groups is possible. The presumed NBHOC origin of emission at ~2 eV also agrees well with the observed increase of its intensity after oxidation at ambient air conditions, as this may facilitate desorption of –OH groups on the surface which leads to stabilization of NBHOC [50].

The above arguments might explain the luminescence specifics of the as-deposited a-Si:Al nanostructure (non-etched Al NWs), which exhibits a single low-intensity PL component centered at 2.2 eV (see Figure 5a). The absence of hollow pores in such a structure implies a considerably smaller effective surface area compared to nanoporous a-Si, and consequently, only relatively minor top-surface oxide contribution shows up in the ~2 eV region of spectra. On the other hand, no observable optical signature of the a-Si matrix in the PL spectrum is likely a result of multiple non-radiative pathways provided by the presence of a dense network of Al NWs.

To get a better insight into the role of native oxide and to single out its luminescence properties, the PL from the nanoporous a-Si was also examined using UV excitation at 325 nm (3.8 eV). In contrast to emission trends observed in the visible range, where the removal of Al-NWs generally increases luminescence yield (quantum efficiency), the PL component in the UV region is observed regardless of the extent of Al NW removal or subsequent oxidation. Figure 6a shows time-lapse PL spectra measured at 10 K of nanoporous a-Si:Al exposed to air for 34 weeks. A series of sharp spectral features can be observed at 3.35, 3.26, 3.18, and 3.0 eV, which appear instable under sustained UV irradiation by the 325 nm excitation source. The stability assessment for two representative emission components, sharp peak (3.18 eV) and broad background (2.84 eV), was performed by de-convoluting these features in each time-lapse spectrum and monitoring evolution of the peak intensities. Figure 6b presents PL intensity as a function of UV irradiation time for the two emission components demonstrating by an order of magnitude larger photodegradation effect (rate) for the sharp features compared to the broad background band centered at 2.84 eV.

The observed PL instability is likely caused by adsorbed species, such as OH, O_2_, H, and H_2_O acting as recombination centers. Desorption of such surface species may also cause a change in the charge state of the surface, changing the band bending at the surface. In turn, this may activate or deactivate different charge states available on the sample surface. Bearing in mind the observed differences in stability, it is reasonable to assume that the broadband at 2.8 eV and the sharp features have a different origin.

**Figure 7:**
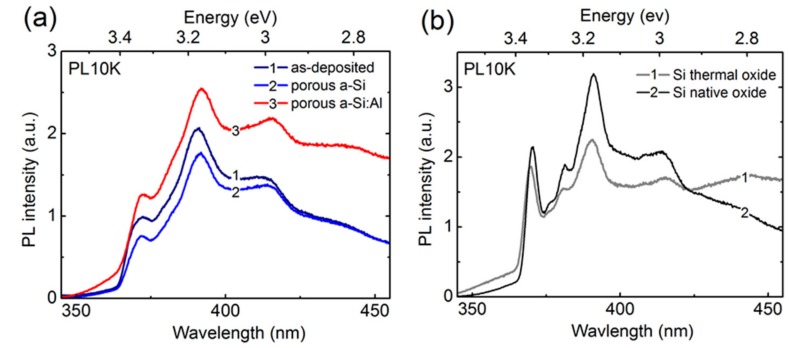
(**a**) Dynamic PL spectra obtained at 10 K from as-deposited a-Si:Al nanostructure, nanoporous a-Si (one-week exposure to air), and porous a-Si:Al (34-week exposure to air), (**b**) monocrystalline Si with 100 nm-thick thermal oxide and native oxide, respectively.

In order to counteract the instability of the sharp features, the spectra presented in Figure 7 were measured under dynamic acquisition conditions, i.e., by continuously moving the probe position over the sample to ensure that the recorded emission originates from yet unexposed area. Figure 7a shows that the intensities of sharp spectral features are similar for all three representative a-Si:Al nanostructures, signifying that their origin is common and most likely related to top-surface. This is further supported by the control measurements of thermal and native silicon oxide on a monocrystalline Si wafer, which show similar features and intensities regardless of oxide layer thickness (Figure 7b).

**Figure 8:**
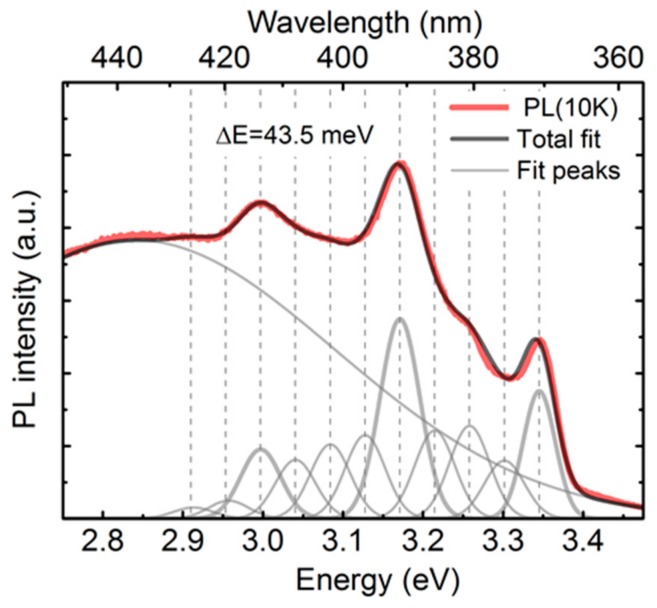
PL spectrum of nanoporous a-Si at 10 K presented alongside with the Gaussian deconvolution components. Vertical markers on the plot indicate equidistant spacing of 43.5 meV.

The unstable spectral features discussed so far are three distinctive sharp peaks positioned at 3.0, 3.16 and 3.34 eV. The equal spacing of 0.17 eV in between these peaks becomes immediately noticeable once the PL spectrum is presented on the photon energy scale, as shown in Figure 8. At closer inspection, one can also observe even finer sub-structure of periodic nature, which is validated by the performed complete Gaussian deconvolution of the spectrum revealing up to 11 modes with an equidistant spacing of 43.5 meV (ω = 350.8 cm^−1^). Such a periodicity suggests the involvement of phonons (vibronic modes), possibly Si-O bending modes that show up in the range 300–400 cm^−1^ according to neutron scattering studies on vitreous silica [51].

With regard to sharp emission features in general, there are several reports for heat-treated silica and alumina systems [52,53,54], as well as various silicon oxide surfaces [55]. Anjiki et al. proposed that the sharp emission is a result of vibrational progressions of the A’^3^Δ_u_ → X^3^Σ_g_^−^ transition of O_2_ molecules [52]. Since the excitation photon energy of 3.8 eV in our experiments appears insufficient for activating A’–X radiative transitions from O_2_, the emission must arise from O_2_ in its already excited state, which may occur during transient cleavage of dioxasilyrane [52]. This is consistent with the decrease of emission intensity under UV exposure, as the surface concentration of potential sources for the excited O_2_ will decrease with irradiation time. Furthermore, another model proposes that the dioxasilirane group on the surface is the origin of the emission at 2.8 eV [56]. However, this would imply the decrease of intensity for the broad background band observed in our experiments which in reality remains stable. It is also important to point out the comparable emission intensity for nanoporous and flat oxidized surfaces (cf. Figure 7a,b), which is in contradiction with earlier reports underlining the necessity of high surface area to observe these features [52,55]. In fact, the similarity in intensity indicates that the source of the sharp features is most likely located at the top surface, and that the increased effective surface area for the nanoporous structure does not contribute significantly in this regard. The possible reason for this could be related to narrow diameter (5-nm) of a hollow channel itself, which either hinders electronic transitions, or prohibits the formation of the responsible defects inside the nanopore. To our knowledge, such sharp features have not been reported for nanostructured Si, whereas two broadband features in the visible and near-UV range are commonly observed for porous Si [13]. Visible emission ranging from 1.1 to 1.9 eV is also reported for Si-nanowires and the observed blue shift is often attributed to quantum confinement effects [57,58]. Here we attribute the shift in visible emission to changing contributions from different luminescence components, i.e., the nanoporous a-Si and the surface oxide.

## 4. Conclusions

Nanoporous a-Si fabricated by selective etching of self-assembled Al NWs in a-Si:Al comprises vertically aligned channels in the a-Si matrix with an oxidized surface. The Al NW removal affects the reflectance and changes the optical appearance of the film to the extent that allows for monitoring and control of the process. Generally, optical properties of nanoporous a-Si are determined by an interplay of bulk, nanosize and surface-related effects, hence understanding of their mutual contributions is imperative for the potential device developments. In the present study, we separate and quantify the role of the bulk and surface effects in optical properties. PL emission in the visible range consists of broadband luminescence centered at 1.9 eV originating from defect centers in the oxide and 1.6 eV emission from radiative recombination within the a-Si core. Sustained (long-term) natural oxidation suppresses the 1.6 eV emission, which is likely due to the reduced amount of elemental Si and an increase in oxidized Si in the nanoporous a-Si. Time-lapse PL reveals sharp spectral features in the range from 3.0 to 3.4 eV, which demonstrate equidistant spacing of 0.17 eV and distinct instability (quenching) under sustained UV irradiation. The observed similarity of emission peaks irrespective of porosity or oxide thickness suggests that their origin is most likely related to the top surface.
